# Systematic observations of enhanced oil recovery and associated changes at carbonate-brine and carbonate-petroleum interfaces

**DOI:** 10.1038/s41598-023-43081-2

**Published:** 2023-10-06

**Authors:** Tianzhu Qin, Paul Fenter, Mohammed AlOtaibi, Subhash Ayirala, Ali Yousef

**Affiliations:** 1 Chemical Sciences and Engineering Division, Argonne National Laboratory, Lemont, USA; 2 EXPEC Advanced Research Center, Saudi Aramco, Dhahran, Saudi Arabia; 3 Present Address: Sigray Corporation, Concord, CA 94520 USA

**Keywords:** Environmental chemistry, Hydrology

## Abstract

Enhanced oil recovery (EOR) from carbonates is obtained by injection of controlled ionic strength brines containing “active ions” (e.g., SO_4_^2−^, Mg^2+^, Ca^2+^). It is generally believed that this occurs through the interaction of the active ions at the carbonate-brine interface (e.g., within a thin brine layer separating the petroleum and the carbonate phases). Here, in-situ observations show how one active ion, SO_4_^2−^, alters behavior at the carbonate-petroleum interface. Displacement of petroleum from initially oil-wet carbonate rocks using brines with variable SO_4_ concentrations systematically changes oil recovery, in situ contact angles, and connectivity of the oil phase, confirming that the active ion alters interactions at the oil/brine/carbonate interface, as expected. Measurements of model calcite-fluid interfaces show that there is no measurable sorption of SO_4_ to carbonate-brine interfaces but reveals that the carbonate-petroleum interface is altered by previous exposure to SO_4_-containing brines. These results suggest that EOR in carbonates is controlled *indirectly* by active ions. We propose that this may be due to a reduced oleophilicity of the carbonate caused by chemical complexation between the active ion and petroleum’s acidic and basic functional groups. This mechanism explains how both anions and cations act as active ions for EOR in carbonates.

Carbonate reservoirs contain about half of the world’s proven petroleum reserves. Waterflooding is a major method to extract oil from geological formations, but this process is inefficient due to the oil-wet preference of carbonate rocks (i.e., with water contact angles > 90°) and the complex rock pore structure. Injection of brines with controlled ionic compositions improves oil displacement efficiency especially for a few “active ions” (i.e., SO_4_^2−^, Mg ^2+^, and Ca^2+^) at elevated concentrations^1–9^, known as enhanced oil recovery (EOR). This behavior is attributed to an increase in water wettability (i.e., hydrophilicity) of the carbonate rocks at increased ion concentrations^2,3^, suggesting that that these active ions alter the interfacial interactions between the oil/brine/rock phases^4^.

Nevertheless, the specific mechanisms underlying this behavior are not well-understood and there are multiple competing conceptual models. A leading explanation is that the improvement of oil displacement efficiency is driven by adsorption of SO_4_^2−^ to the rock/brine interface leading to double-layer expansion of thin water films on rock surfaces^1,5–9^. It is also thought that adsorption of SO_4_^2−^ can enhance the co-adsorption of Ca^2+^ and Mg^2+^ at the carbonate mineral surfaces and disrupt the interactions between carboxylic groups of the petroleum and the carbonate surface^3,5–8^. This cooperative behavior is expected to increase with increasing temperatures, consistent with known improvements of oil displacement at elevated temperatures^3,9,10^. The sensitivity of oil-rock interactions to the presence of these active ions has been observed using *in-situ* observations of oil–water-rock contact angles observed in recent X-ray tomography studies^11,12^. Notably, injection water with high sulfate concentrations was seen to alter the wettability of pore surfaces within minutes of exposure^13,14^.

Here we report systematic experimental observations of petroleum/brine/carbonate interfaces that reveal a new understanding of the systematic controls of brine composition (specifically SO_4_^2−^ concentration) on petroleum production. First, we confirm that elevated SO_4_^2−^ electrolyte ion concentrations lead to enhanced oil recovery through operando measurements of core flooding of natural carbonate rocks at elevated temperatures representative of the subsurface. Second, we perform spontaneous imbibition studies as a function of [SO_4_] to reveal significant changes in oil recovery, in-situ observed water contact angles, and measurements of oil-phase connectivity. These behaviors are correlated and follow sigmoidal responses as a function of [SO_4_]. Third, we perform molecular-scale observations of model calcite single crystal surfaces interacting with the brines and petroleum. These studies reveal that there is no measurable affinity of SO_4_^2−^ at the carbonate-brine interface (e.g., adsorption, incorporation). Instead, we find that the carbonate-petroleum interface is altered due to previous exposure to sulfate-containing brines. Together, these results suggest that EOR is controlled by reduced oleophilicity in the presence of SO_4_ (i.e., weakening interactions at the carbonate-petroleum interface) rather than increased hydrophilicity of the carbonate-brine interface.

## Results

### Operando observations of enhanced oil recovery with SO_4_ brines

Experimental details and procedures are described in the “Materials and Methods” section. Core flooding and spontaneous imbibition tests were performed using natural carbonate rock samples obtained from a carbonate oil field. We focus on the effect of SO_4_ ions by using two end-member synthetic brines and their mixtures (Table 1) to probe the role of SO_4_ concentrations at fixed salinity. Tailored Water 1 (“TW1”) consists predominantly of Na^+^ and Cl^-^ along with [SO_4_] = 429 ppm and lesser amounts of Mg^2+^ and Ca^2+^ at an ionic strength of 0.12 M. Tailored Water 2 (“TW2”) consists of Na_2_SO_4_ at the same ionic strength, with [SO_4_] = 3896 ppm.

**Table 1:** Compositions and characteristics of synthetic brines.

	Formation water (ppm)	Tailored water 1 (ppm)	Tailored water 2 (ppm)
Sodium	59,491	1830	1865
Calcium	19,040	65	0
Magnesium	2439	211	0
Sulfate	350	429	3896
Chloride	132,060	3220	0
Bicarbonate	354	12	0
TDS	213,734	5767	5761
Ionic strength (Mol/L)	4.318	0.115	0.123

**Figure 1:**
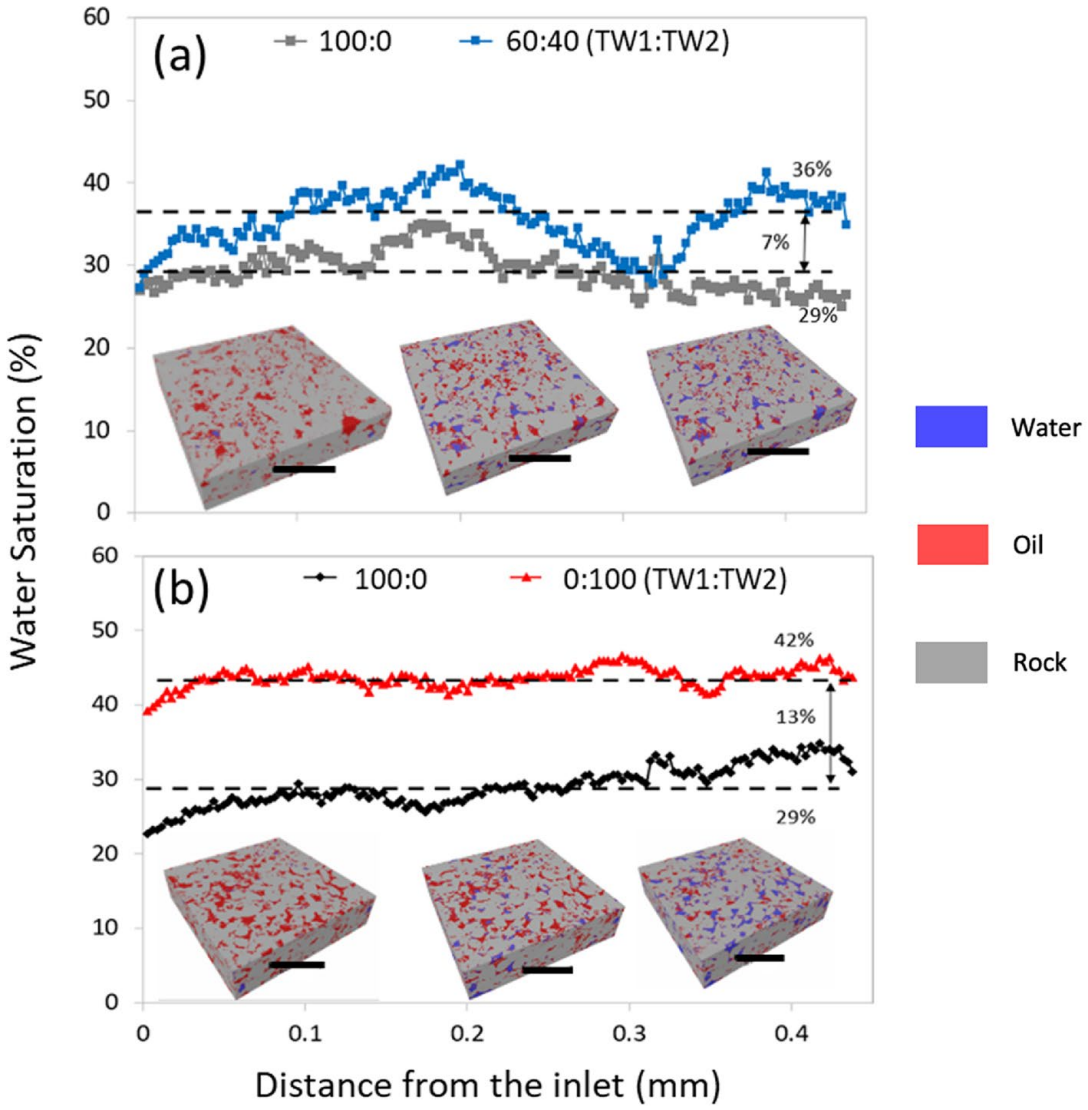
Water saturation profiles in core flooding measurements at 90 °C are shown for (**a**) Sample A after displacing petroleum with 10 PV of TW1, and after 10 PV of a 60:40 TW1/TW2 mixture, and (**b**) Sample B after oil displacement with TW1 then TW2. Cross sectional images of the rock, obtained by XCT, in contact with oil and after displacement using each of the two brines is shown (inset, scale bars indicate 1 mm). (XCT images are segmented to show water and oil as blue and red regions, respectively, while the carbonate matrix is shown in grey).

The core flooding tests were performed with two rock specimens (Samples A and B) that were previously saturated by petroleum (Fig. 1). The displacement of petroleum from the oil saturated rock was performed at 90 °C and P = 2.5 bars, representing the conditions of the subsurface. Operando images by synchrotron CT show the initially petroleum-saturated rock and after the petroleum was displaced by 10 pore volumes (PV) of TW1. Both samples were found to have an equivalent oil recovery of 29%. A second core displacement measurement for Sample A using 10 PV of a 60:40 mixture of TW1:TW2 led to an additional 7% oil recovery, or a net oil recovery of 36%. In contrast, the second core displacement for Sample B using the TW2 brine had an additional 13% oil recovery, double that observed for the TW1:TW2 mixture, for a net oil recovery of 42%, directly confirming that SO_4_ induces EOR.

### Systematic controls over enhanced oil recovery by SO_4_

**Figure 2:**
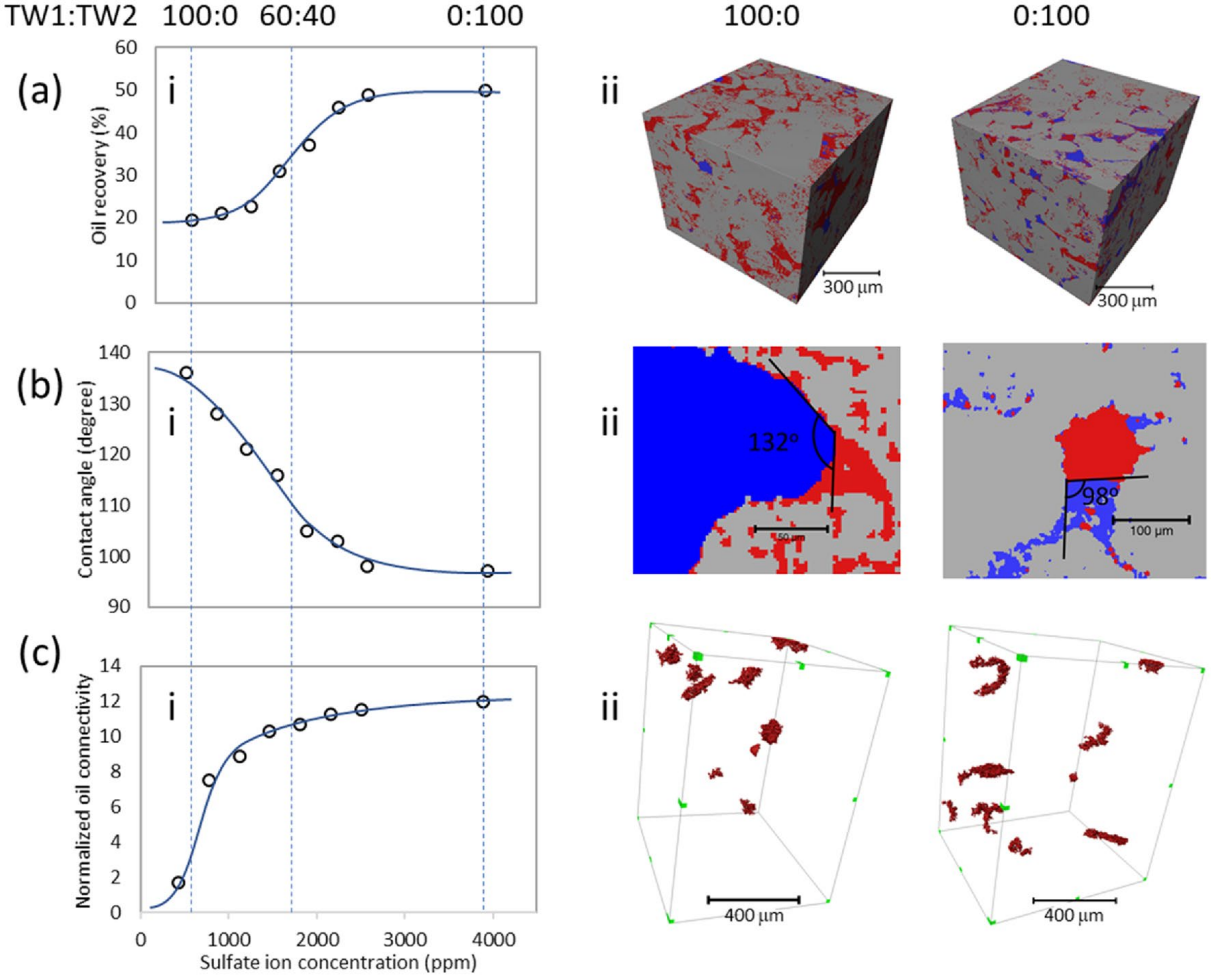
Systematic changes in oil–water-calcite interactions vs. [SO_4_] showing changes in (**a**) average oil recovery, (**b**) oil-brine-calcite contact angle, and (**c**) oil connectivity as a function of brine sulfate concentration obtained through spontaneous imbibition tests with TW1 and TW2 and their mixtures. (Images in a-ii and b-ii are segmented XCT images where water and oil are shown as blue and red regions, respectively, while the carbonate matrix is shown in grey). Each data point is obtained on separately prepared natural samples from the same core section. The 2nd and 3rd columns provide a visualization of each quantity using TW1 (100:0) and TW2 (0:100), respectively. Images of oil connectivity (c-ii), the 10 largest oil droplets in the rock specimen are visualized to illustrate the change in droplet compactness.

Insights into the chemical controls over enhanced oil recovery were obtained through systematic *in-situ* observations of spontaneous imbibition using separately prepared natural rock samples as a function of [SO_4_] (Fig. 2). Aged oil-saturated rock samples were immersed into brines and equilibrated for one day at 90 °C for a range of sulfate ion concentrations at fixed ionic strength (using the TW1 and TW2 brines, and their mixtures). The fluid occupancy in the pores of these rock samples were visualized in three-dimensions (3D) using synchrotron X-ray microtomography (Fig. 2aii). At lower sulfate concentrations, the brine phase preferred to stay in the large pores due to the lower negative threshold capillary pressure in the pores with larger radius. With increasing [SO_4_], the water saturation inside the pores increased and the brine phase invaded the smaller pores that have higher negative capillary pressures. Averaged over the rock specimens (Fig. 2ai), the water saturation in the carbonate rock samples showed a significant (> twofold) increase in oil displacement efficiency at the highest [SO_4_], consistent with the core flooding observations (Fig. 1), with a sigmoidal shape vs. [SO_4_].

Simultaneous with the increase of average oil recovery, the water-carbonate contact angle decreases from 132° to 98° showing that the carbonate surfaces change from being oil-wet to neutral-wet with increasing sulfate concentrations (Fig. 2b). The wettability alteration of the pore surfaces reached a plateau, stabilizing for sulfate ion concentration above 2500 ppm, consistent with the observed changes in average water saturation. Oil channels play an important role in the oil displacement. Visualization of the oil channels inside the porous carbonate rock (Fig. 2bii) shows that the oil occupies the center of the pores at high oil saturation but stayed in the pore corners and formed flow channels in the pores at low oil saturation. The connectivity of these channels was quantified using the normalized Euler number of the oil phase following the approach in previous works^14–16^ (Fig. 2c). Briefly, the Euler number of oil was determined by calculating the differences between isolated oil ganglia and the oil loops and normalized by the Euler number of the pore space. The normalized Euler number is small at low sulfate concentrations indicating that there are few oil channels in the porous medium in the absence of sulfate, and it increased with sulfate concentration indicating that oil channels become more abundant, reaching a plateau at the highest concentrations (Fig. 2cii).

**Figure 3:**
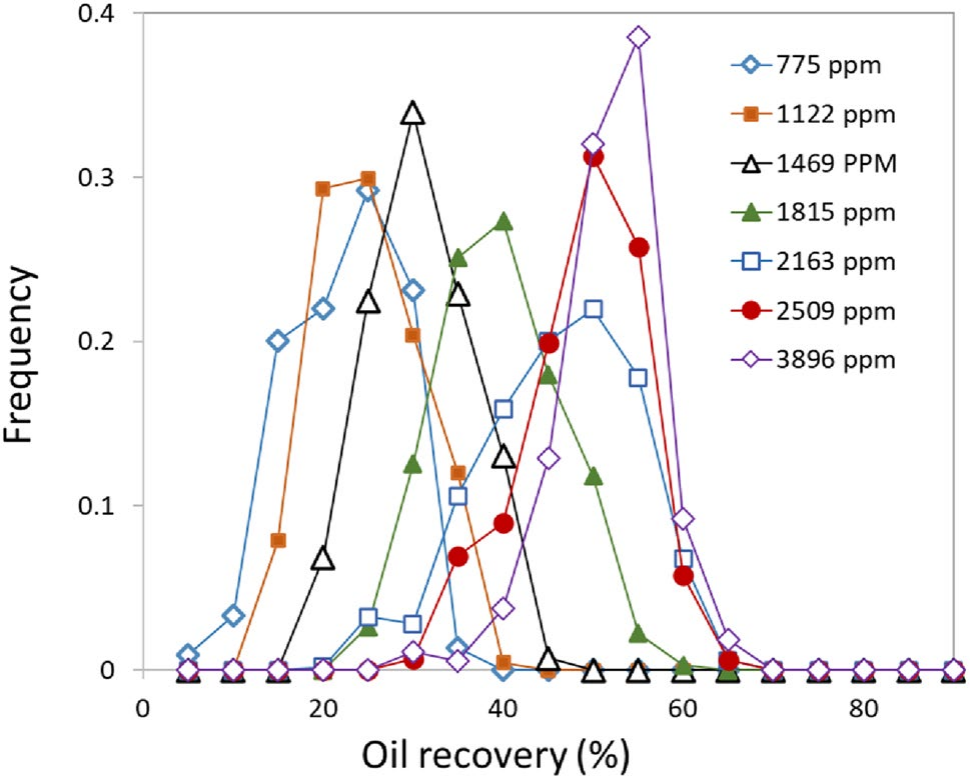
Systematic changes to oil recovery distributions along the mixing line of TW1 and TW2 (during spontaneous imbibition tests) showing a uniform increase in oil recovery across the sample at increasing [SO_4_] (in units of ppm).

This observed changes in oil connectivity are strongly correlated with the associated changes in wettability alteration and water saturation, suggesting that all three behaviors are controlled by interfacial interactions at the oil/brine/carbonate interfaces. Further evidence for this inference is obtained by plotting a histogram of fractional oil recovery for all virtual cross sections within the sample (Fig. 3) as a function of brine composition. This result shows that the histogram of observed oil recovery has a well-defined bell-shaped distribution. Notably, this distribution is peaked at ~ 20% for the low-SO_4_ brines and the center of this distribution shifts to ~ 50% at the highest SO_4_ concentrations with only minimal broadening for intermediate concentrations. This observation indicates that the increase in water wetness and displacement efficiency occurs nearly uniformly throughout the rock sample.

**Figure 4:**
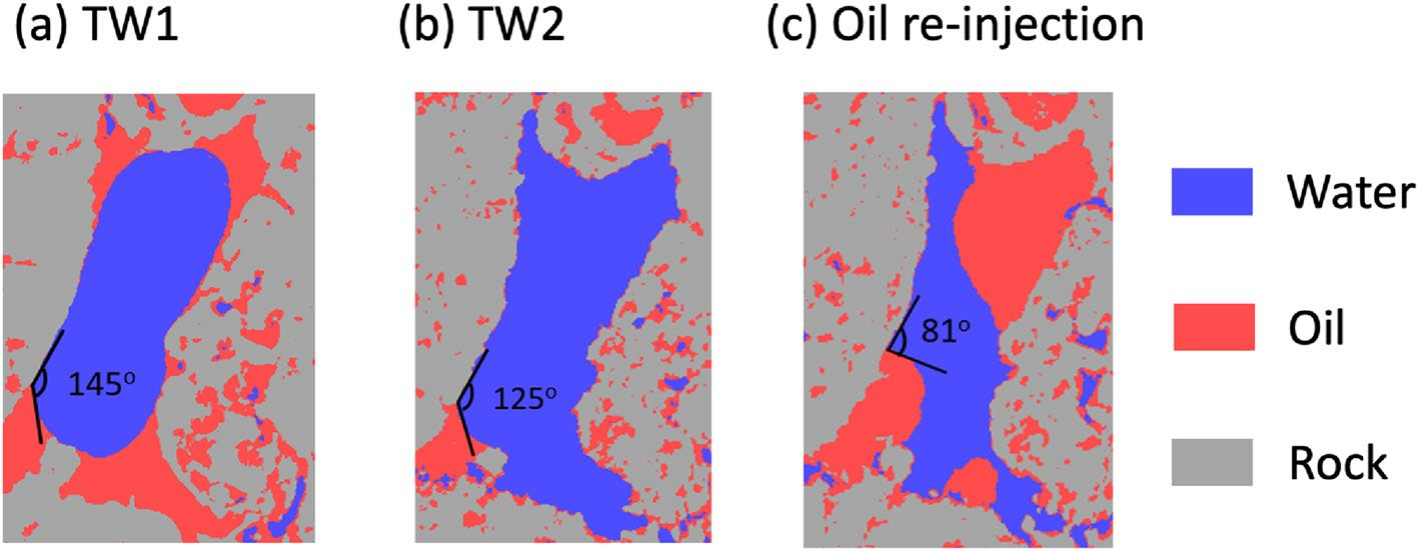
In-situ changes to the oil-brine-calcite contact angles at 25 °C in a single pore during core flooding (**a**) after displacement of petroleum by TW1 (with a contact angle of 145°), (**b**) after the TW1 brine is displaced by TW2 reducing the contact angle to 125° while the three-phase contact point is unchanged. (**c**) Re-injection of petroleum to move the three-phase contact point and leads to a decrease in the contact angle to 81°. (Here, water and oil are shown as blue and red regions, respectively, while the carbonate matrix is shown in grey).

Further insight into the role of SO_4_ concentrations in controlling the observed contact angles is shown in core flooding measurements (Fig. 4), where the same pore region was imaged sequentially after multiple fluid displacements at 25 °C. After petroleum was displaced by TW1, the pore shows a large contact angle (~ 145°) (Fig. 4a) that is expected for the oil-wet behavior found in TW1 (Fig. 2b). The image of the same pore after the TW2 displaced the TW1 brine (while maintaining the same oil–water contact point) shows that the contact angle decreased to 125° (Fig. 4b). This change confirms the sensitivity of the contact angle to the choice of brine, but this value is significantly larger than that observed in spontaneous imbibition studies with TW2 (Fig. 2b). That is, the impact of SO_4_ on EOR is not solely due to its interaction at the carbonate-brine interface. Finally, petroleum oil was re-injected to move the oil–water-carbonate contact point to a position that previously was directly exposed to TW2, at which point the observed contact angle decreased to 81° (Fig. 4c) consistent with the observed value obtained during spontaneous imbibition tests (Fig. 2b). This sensitivity of the contact angle to the location of the three-phase contact point immediately implies that SO_4_-induced changes to the *petroleum-carbonate interface* is a significant contribution to wettability alteration.

### Molecular-scale observations of petroleum-brine-calcite interfaces

**Figure 5:**
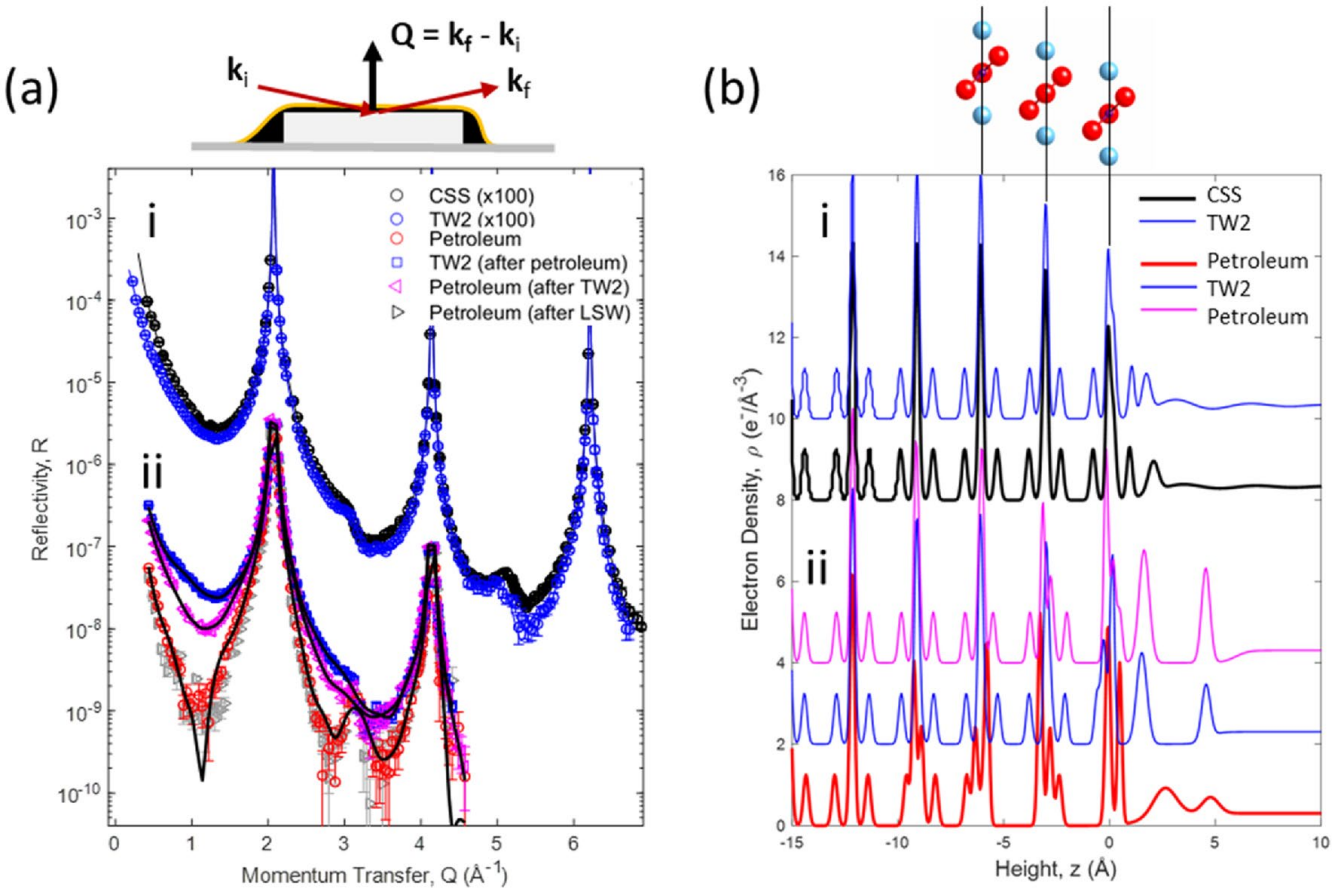
Molecular Scale Characterization of Carbonate Interfaces (**a**) XR of calcite single crystals in contact with (ai) calcite saturated solution (CSS) and the TW2 brine (40 mM Na_2_SO_4_), and (aii) a single calcite sample that was exposed sequentially to natural petroleum oil (red circles), then the TW2 brine (blue squares), and petroleum (magenta triangle). (**b**) Derived density profiles for each data set shown in (**a**). These results show that the calcite-CSS and calcite-TW2 interfaces are equivalent, and that the original calcite-petroleum interfacial structure differs from that observed after the sample was exposed to the TW2 brine.

Mechanistic insights into the role of SO_4_ in mediating the interactions at brine/oil/carbonate interfaces that govern EOR are obtained through direct molecular-scale observations of flat single crystal calcite surfaces using in-situ X-ray reflectivity (XR). We first compare the structure of calcite single crystal surfaces in the TW2 brine with previous observations of calcite in a calcite-saturated aqueous solution^17,18^ (Fig. 5ai) to probe the specific affinity of SO_4_ to the carbonate surface since. Sensitivity of XR to adsorbed SO_4_ derives from its ~ fivefold higher X-ray scattering factor (Σ_i_ Z_i_ = 48) as compared to H_2_O (Σ_i_ Z_i_ = 10). The XR signals are visibly similar for these two conditions, as are the laterally averaged density profiles obtained from the best-fit interfacial models (where the effect of extrinsic factors such as surface roughness and fluid layer thickness are removed). Both of these calcite-brine interfaces are described by an interfacial water structure with two adsorbed water layers, and slight distortions to the calcite structure that extends ~ 3 layers below the calcite surface (Fig. 5bi) as described previously^17,18^. (Optimized structural parameters that describe these structures are listed in Supplemental Table 1). These interfacial structural distortions derive from the separate vertical displacements of the Ca^2+^ and CO_3_^2−^ ions and changes in the angle of the CO_3_ plane. The lack of any significant enhancements in the density profile at the calcite-brine interface reveals there is no measurable sorption of SO_4_ to the calcite surface in the TW2 brine, the brine composition for which wettability alteration is maximized (Fig. 2). This observation contradicts a key assumption that wettability alteration is driven by the electrostatic attraction of SO_4_^2-^ ions to the carbonate-water interface^1,5–9^. This lack of SO_4_^2−^ adsorption in these measurements also is consistent with recent studies showing that there is little or no net charge at the calcite-water interface (using Rb^+^ as a probe ion)^19^.

Measurements of the calcite-petroleum interface before and after exposure to brines provides additional insights into these complex interactions. The role of SO_4_ in EOR is revealed by comparing XR results from a calcite crystal after sequential exposures to petroleum, TW2 and petroleum (Fig. 5bii). The calcite-petroleum interface is very different from that seen in brines^20^. The XR signals from the calcite-petroleum interface (red circles, Fig. 5aii) show a ~ 50-fold reduction in the XR signal near Q = 1.1 Å^−1^ with respect to the calcite-water interface. The derived density profile (red line, Fig. 5bii) reveals that calcite-petroleum interface structure has significantly larger structural rearrangements than present for the calcite-brine interface, along with the presence of a high-density layer adsorbed above the calcite surface, and significant surface roughness, all suggesting strong chemical interactions between calcite and petroleum. (Optimized structural parameters that describe these structures are listed in Supplemental Table 2).

The calcite-TW2 interface after displacement of petroleum (blue circles, Fig. 5bii) has XR signals that are broadly similar to the calcite-brine interface (Figs. 5aii) and characterized by small interfacial displacements (as seen previously^20^). The calcite-petroleum interface after displacement of the TW2 brine (magenta triangles, Figs. 5aii) retains many of the general characteristics of the calcite-brine interface rather than adopting the characteristics of the calcite-petroleum interface (red circles, Fig. 5aii). For instance, the XR signals show only a ~ threefold reduction in the mid-zone (i.e., near Q = 1.1 Å^−1^) with respect to the calcite-water interface (black circles, Figs. 5ai), as compared to the 50-fold reduction observed in petroleum (Fig. 5aii). This observation is distinct from previous results that demonstrated that the XR data, and the associated structure of the calcite-petroleum interface, are not influenced by prior exposure to low-[SO_4_] brines. For example, XR data of the calcite-petroleum interface after exposure to a high salinity brine with low SO_4_ content representing formation water (grey triangles, Fig. 5aii; previously reported in Ref.^20^) are indistinguishable from those of the calcite-petroleum interface without prior exposure to any brine (red circles, Fig. 5aii).

That is, exposure of the carbonate surface to high concentration SO_4_ brines alters the structure of the subsequent calcite-petroleum interface. The derived interfacial density profile (magenta line, Fig. 5bii) reveals that the calcite-petroleum interface after exposure to TW2 brine has much smaller sub-surface interfacial displacements within the top four Ca-CO_3_ layers (magenta lines, Fig. 5bii) than the calcite-petroleum without prior exposure to the TW2 brine. We have previously discussed how the magnitude of structural displacements in the calcite surface can be thought of as a measure to probe the chemical interactions between calcite and a fluid^20^. Therefore, the smaller subsurface displacements observed in Fig. 5 indicate that prior exposure to SO_4_-containing brines effectively weakens calcite-petroleum interactions.

## Discussion

The present results demonstrate that SO_4_ ions dissolved in brines influence oil-carbonate interactions, including significant enhancements in observed oil recovery that saturates at [SO_4_] > 2500 ppm. The efficacy of these behaviors at the conditions of the subsurface reservoir provides a benchmark for target brine concentrations for oil production. The systematic change in EOR performance is associated with changes to the wettability of the carbonate rock and associated changes in oil connectivity (as measured by the normalized Euler number) which is observed as the development of oil channels. The development of continuous oil channels could be explained by increasing viscoelasticity of oil/brine interfaces and thereby less interfacial pinch-off with increasing SO_4_ concentrations^21–23^. The high consistency of behavior between natural carbonate rock samples and model calcite interfaces suggests that our observations reflect the intrinsic characteristics of the petroleum-carbonate interactions and that these observations are not obscured by significant differences in the extrinsic properties of these systems (e.g., such as surface roughness, curvature, impurities).

The observed changes in oil-brine-carbonate contact angle, θ, with increased [SO_4_] (Fig. 2) unambiguously confirms that SO_4_ ions alter the relative interfacial energies (γ) of the carbonate-brine (c-b), carbonate-oil (c-o) and oil-brine (o-b) interfaces as described by the Young’s equation, γ_c−b_ = γ_c-o_ + γ_o−b_ cos(θ). It is widely assumed that these changes derive primarily from direct SO_4_ adsorption at the calcite-brine interface^1,3,5–9^ (as might reasonably be expected since SO_4_ is dissolved in brine),

However, the present observations challenge some of the widely accepted mechanisms associated with EOR. For example, our observations using model calcite surfaces show no measurable surface activity of SO_4_ at this interface (Fig. 5ai,bi) nor the presence of a thin brine wetting layer (which is needed to mediate the proposed SO_4_-carbonate interactions at the petroleum-carbonate interface). In the absence of any changes at the carbonate-brine interface associated with increased [SO_4_], the Young’s equation implies that the petroleum-brine-carbonate contact angle might be controlled instead by interactions at either the carbonate-petroleum or petroleum brine interfaces.

The results directly reveal that the interfacial structure of the petroleum-carbonate interface, as seen using XR with model calcite single crystal surfaces (Fig. 5), is altered due to previous exposure to high [SO_4_] brines. Notably, the strong interactions at the petroleum-carbonate interface in the absence of SO_4_ (indicated by its oil-wet behavior, Fig. 4, and the large structural displacements within the top calcite surface layers, Fig. 5) is reduced by prior exposure to the SO_4_ brines. This suggests that SO_4_ alters the petroleum-brine-carbonate interactions and alters the observed wetting behavior by *reducing* the initially strong petroleum-carbonate interactions (i.e., reduced oleophilicity) rather than to *increasing* the affinity of brines to the carbonate surface (i.e., enhanced hydrophilicity) as is usually assumed.

It is challenging, given the high complexity of petroleum, to identify a single molecular-scale mechanism that explains the changes in carbonate wettability and enhancements in oil recovery associated with the presence of SO_4_. The present observations and constraints, especially the observation that SO_4_ alters the carbonate-petroleum interface without the presence of a thin brine wetting layer, suggests that this behavior may be controlled *indirectly* rather than by simple adsorption of SO_4_ from the brine phase. A hypothesis that is suggested by these results is that the changes in petroleum wetting may be controlled by chemical complexation between the SO_4_ anion with positively charged quaternary ammonium basic functional groups within petroleum^24,25^. This complexation could inhibit interactions between petroleum with the carbonate surface, similar to what has been observed for studies of the interaction of model oils (e.g., stearic acid)^26,27^ thereby weakening the petroleum-carbonate interaction and relieving the structural interfacial structural strains that we observe at the petroleum-carbonate interactions in the absence of SO_4_. This mechanism might act either indirectly (i.e., within the petroleum phase by solution complexation) or at the carbonate interface (i.e., if the quaternary ammonium basic functional groups from the petroleum themselves are adsorbed to the calcite surface). Furthermore, it is possible that the incorporation of partially hydrated SO_4_ ions may enable water molecules to coordinate with the carbonate surface thereby reducing the strong structural displacements associated with petroleum-carbonate interactions. While these ideas will need to be tested by additional studies, this proposed mechanism is attractive as it also provides an explanation for how cations (e.g., Ca^2+^ and Mg^2+^) can also act as “active ions” for EOR, even though their interactions with carbonate surfaces should differ significantly from that of SO_4_ (due to their different size and sign of charge). Since petroleum has both acidic and basic functional groups, the influence of the interaction of active cations (e.g., Ca^2+^, Mg^2+^) with the acidic functional groups in petroleum (e.g., carboxylic acids) might be similar to that of SO_4_^2−^ with the basic functional groups. Since TBN >  > TAN (1.1 mg KOH/g vs. 0.085 mg KOH/g) for crude oil, there is a greater possibility for operability of this mechanism^28^.

## Materials and methods

### Fluids

Natural petroleum oil from a carbonate reservoir^16^ was amended with di-iododecane to provide contrast in the X-ray CT images with respect to the aqueous phase^14^. Synthetic brines (synthesized formation brine, TailoredWater1 (TW1), and TailoredWater2 (TW2)) composed of different ions were applied in this study. TW1 and TW2 were mixed at different ratios (e.g., 25:75, 50:50, 75:25) to investigate the impact of ion types and concentrations on the oil recovery. The brine solutions ion compositions are tabulated in Table 1.

Natural crude oil from a carbonate reservoir was centrifuged at 1000 rpm and filtered with 0.5 um filter to remove any debris. The crude oil was then doped with 14 wt.% di-iododecane to increase the contrast between oil and brine phases in the microtomographic images. Total base number (TBN) of crude oil is 1.1 mg KOH/g, while the total acid number (TAN) is < 0.085 mg KOH/g^28^. 

### Rock sample preparation

The detailed method for preparing carbonate rock samples for the core-flooding and spontaneous imbibition tests was described in a previous manuscript^14^. Briefly, carbonate rock samples were selected from carbonate oil reservoir rocks, cut into plates using a diamond saw, and cleaned with toluene/methanol mixtures at elevated temperature to restore the water-wetness of the rocks. The pore size distributions show that the rock is heterogenous with respect to pore size distribution (Fig. 1a, inset), with 25% of small pores (r < 80 μm), 50% median sized pores (80 < r < 120 μm), and 25% large pores (r > 120 μm), but each rock sample had similar pore topologies as measured by their cumulative pore size distribution. (Fig. 1b, inset).

The rock substrates were then cut into cubical rock samples 2 mm in length vacuumed and saturated with formation brine, and then saturated in natural petroleum oil by centrifuging with doped crude oil at 1000 rpm for 20 min and aged at 90 °C for seven days. Samples were then carefully transferred into closed polycarbonate (PC) cells for measurements. The rock samples were immersed in brine solutions with different ion composition at 90 °C for one day before being visualized by synchrotron-based X-ray microtomography.

Core-flooding experiments were carried out on two rock specimens to confirm the correlation between elevated SO_4_^2-^ electrolyte ion concentrations and improved oil recovery at 90 °C. The detailed experimental procedures can be found in the prior studies^29,30^. Furthermore, an additional core sample was utilized to understand the impact of varying SO_4_^2−^ concentrations on the contact angles. Subsequent to the core-flooding experiments described in previous study^14^, oil was re-introduced at the flow rate of 0.02 cc/min for 30 s and then visualized by X-ray microtomography.

### Methods for CT

Rock samples were visualized in 7-BM and 13-BM using “white” and “pink” X-ray beams with energy ranges from 10 to 170 keV and 4.5 to 100 keV, respectively and an exposure time of 45 secs at the Advanced Photon Source. The tomographic data collected from 13-BM were reconstructed in the use station using IDL software. The tomographic data from 7-BM on the other hand were reconstructed using Tomopy software package and the Bebop in the Laboratory Computing Resource Center in Argonne National Laboratory to expedite the reconstruction of the massive amount of data. The centers of rotations were calculated automatically by Tomopy and evaluated manually after reconstruction processes. The reconstructed tomographic images were transformed to remove the background. White noise was removed using the non-local means filter to improve the signal/noise ratio. The edges between oil/brine/rock phases were detected according to the local gradient of the intensity profiles of the images. The representative regions of different phases were selected according to their intensities and marked as the seeds. The seeds for different phases were then expanded until they meet at the edges of each phase**.** The oil recovery of rock sample of each slice was calculated, and then used for evaluating the oil recovery distribution along the rock samples.

### Calcite surfaces

Natural calcite single crystal surfaces were prepared by cutting a large calcite rhomb (~ 50 mm across) with a diamond saw to create calcite rod with ~ 1/2 cm^2^ cross-section, whose axis is perpendicular to the calcite (104) surface. Calcite crystals approximately 2 mm thick were then cleaved from the rod using a razor blade and the crystal was promptly placed in solution of interest to equilibrate before being placed in a “thin-film” cell for X-ray measurements.

### Methods for XR of calcite surfaces

X-ray reflectivity measurements were performed at sector 33-BM-C at the Advanced Photon Source at Argonne National Laboratory. The measurements were performed with a monochromatic photon energy of 21 keV and a beam flux of ~ 5 × 10^11^ photons/sec, using a “thin film” cell^31^ in which a calcite sample is held in contact with a thin fluid layer with a Kapton membrane that holds a ≥ 2 μm-thick fluid film in place.

The XR technique^31–33^ probes interfacial structure through the variation of the specular (i.e., mirror-like) reflectivity, R(Q) as a function of momentum transfer, Q which is related to the angle of incidence (θ, with respect to the surface plane) through the relation: Q ≡ (4π/λ)sin(θ). The measured XR signal is related directly to the laterally averaged electron density profile at the solid–liquid interface, ρ(z), as a function of the height, z, above and below the interface through the relation:

R(Q) = (4πr_e_/A_UC_ Q)^2^ | Σ_j_ f_j_(Q) O_j_ exp(iQz_j_) exp[−1/2(Qσ_j_)^2^] |^2^,

where r_e_ = 2.818 × 10^–5^ Å, and A_UC_ = 20.2 Å^2^ is the surface unit cell area of the calcite(104) surface. The interfacial structure is obtained through least-squares fitting of the XR data using molecular-scale models that include the substrate mineral bulk and surface structure, the presence of any adsorbed species from the fluid as defined by the height, z_j_, occupation, O_j_, and root-mean square distribution, σ_j_, for each atom, j, at the interface and the average surface roughness. The only unknown in the reflectivity signal derives from the structure at the interface, including the top few calcite surface layers and the near-surface adsorbed layers.

As described previously^20^, caution is needed in interpreting the optimized structural models. The model implicitly assumes that the interfacial structure is laterally uniform (i.e., having the same molecular-scale structure within each surface unit cell) and that it can be described by a unique substrate structural distortion, a few adsorbed species at the calcite surface, along with a bulk-like fluid above the surface. The composition of the petroleum oil, unlike simple brine solutions, is highly complex, with hundreds of individual molecular components, each of which may behave differently. Furthermore, the inferred interfacial structural distortion is described by large structural changes that are likely to be controlled by the specific interaction with adsorbed species that may include large molecules and oligomers. Therefore, the actual interfacial structure is likely to be laterally heterogeneous at the molecular-scale. As such, the derived structural models to describe calcite-petroleum interfaces should not be taken literally (i.e., as indicating the actual location of each atom and molecule), but instead are best interpreted as representing the effective (laterally averaged) interfacial structure.

### Supplementary Information

Supplementary Tables.

## Data Availability

Data from this manuscript will be made available upon requests sent to Paul Fenter (fenter@anl.gov).

## References

[1] Awolayo A, Sarma H, AlSumaiti A (2016). An experimental investigation into the impact of sulfate ions in smart water to improve oil recovery in carbonate reservoirs. Transp. Porous Med..

[2] Saeedi Dehaghani AH, Badizad MH (2019). Impact of ionic composition on modulating wetting preference of calcite surface: Implication for chemically tuned water flooding. Colloids Surf. A Physicochem. Eng. Asp..

[3] Zhang P, Austad T (2006). Wettability and oil recovery from carbonates: Effects of temperature and potential determining ions. Colloids Surf. A.

[4] Fathi SJ, Austad T, Strand S (2011). Water-based enhanced oil recovery (EOR) by "Smart Water": Optimal ionic composition for EOR in carbonates. Energy Fuels.

[5] Austad T, Shariatpanahi SF, Strand S, Black CJJ, Webb KJ (2012). Conditions for a low-salinity enhanced oil recovery (EOR) effect in carbonate oil reservoirs. Energy Fuels.

[6] D. J. Ligthelm, J. Gronsveld, J. Hofman, N. Brussee, F. Marcelis, and H. van der Linde, *Novel Waterflooding Strategy By Manipulation Of Injection Brine Composition*, Soc Petrol Eng J, SPE ( 2009).

[7] RezaeiDoust A, Puntervold T, Strand S, Austad T (2009). Smart water as wettability modifier in carbonate and sandstone: A discussion of similarities/differences in the chemical mechanisms. Energy Fuels.

[8] Strand S, Austad T, Puntervold T, Hognesen EJ, Olsen M, Barstad SMF (2008). "Smart Water" for oil recovery from fractured limestone: A preliminary study. Energy Fuels.

[9] Zhang P, Tweheyo MT, Austad T (2007). Wettability alteration and improved oil recovery by spontaneous imbibition of seawater into chalk: Impact of the potential determining ions Ca^2+^, Mg^2+^, and SO_4_^2−^. Colloids Surf. A.

[10] Strand S, Hognesen EJ, Austad T (2006). Wettability alteration of carbonates—Effects of potential determining ions (Ca^2+^ and SO_4_^2-^) and temperature. Colloids Surf Physicochem. Eng. Aspects.

[11] Khishvand M, Alizadeh AH, Kohshour IO, Piri M, Prasad RS (2017). In situ characterization of wettability alteration and displacement mechanisms governing recovery enhancement due to low-salinity waterflooding. Water Resour. Res..

[12] Xie Y, Khishvand M, Piri M (2020). Impact of connate brine chemistry on in situ wettability and oil recovery: Pore-scale experimental investigation. Energy Fuels.

[13] T. Qin, P. Fenter, M. AlOtaibi, S. Ayirala, and A. AlYousef, *Microscale Investigation of Dynamic Wettability Alteration Effect on Oil Displacement by Smart Waterflooding Using Synchrotron-Based Microtomography*, Abu Dhabi International Petroleum Exhibition & Conference (2020).

[14] Qin TZ, Fenter P, AlOtaibi M, Ayirala S, AlYousef A (2021). Pore-scale oil connectivity and displacement by controlled-ionic-composition waterflooding using synchrotron X-ray microtomography. SPE J..

[15] Herring AL, Harper EJ, Andersson L, Sheppard A, Bay BK, Wildenschild D (2013). Effect of fluid topology on residual nonwetting phase trapping: Implications for geologic CO2 sequestration. Adv. Water Resour..

[16] Rucker M (2015). From connected pathway flow to ganglion dynamics. Geophys. Res. Lett..

[17] Fenter P, Kerisit S, Raiteri P, Gale JD (2013). Is the calcite-water interface understood? Direct comparisons of molecular dynamics simulations with specular X-ray reflectivity data. J. Phys. Chem. C.

[18] Fenter P, Sturchio NC (2012). Calcite (104)-water interface structure, revisited. Geochim. Cosmochim. Acta.

[19] Lee SS, Heberling F, Sturchio NC, Eng PJ, Fenter P (2016). Surface charge of the calcite (104) terrace measured by Rb^+^ adsorption in aqueous solutions using resonant anomalous X-ray reflectivity. J. Phys. Chem. C.

[20] Fenter P, Qin TZ, Lee SS, AlOtaibi MB, Ayirala S, Yousef AA (2020). Molecular-scale origins of wettability at petroleum-brine-carbonate interfaces. Sci. Rep..

[21] Ayirala SC, Al-Yousef AA, Li ZL, Xu ZH (2018). Water ion interactions at crude-oil/water interface and their implications for smart waterflooding in carbonates. SPE J..

[22] Bidhendi MM, Garcia-Olvera G, Morin B, Oakey JS, Alvarado V (2018). Interfacial viscoelasticity of crude oil/brine: An alternative enhanced-oil-recovery mechanism in smart waterflooding. SPE J..

[23] Chavez-Miyauchi TE, Firoozabadi A, Fuller GG (2016). Nonmonotonic elasticity of the crude oil-brine interface in relation to improved oil recovery. Langmuir.

[24] Bonto M, Eftekhari AA, Nick HM (2019). An overview of the oil-brine interfacial behavior and a new surface complexation model. Sci. Rep..

[25] M. P. Yutkin, K. M. Kaprielova, S. Kamireddy, A. Gmira, S. C. Ayirala, C. J. Radke, and T. W. Patzek, in *SPE Improved Oil Recovery Conference, Virtual, April 2022.* 2022), pp. 209389.

[26] Gomari KAR, Hamouda AA, Denoyel R (2006). Influence of sulfate ions on the interaction between fatty acids and calcite surface. Colloids Surf. A.

[27] Tabrizy VA, Hamouda AA, Denoyel R (2011). Influence of magnesium and sulfate ions on wettability alteration of calcite. Quartz Kaolinite Surf. Energy Anal. Energy Fuels.

[28] Rao A (2022). Formation and stability of heterogeneous organo-ionic surface layers on geological carbonates. Energy Fuels.

[29] Yousef AA, Al-Saleh S, Al-Kaabi A, Al-Jawfi M (2011). Laboratory investigation of the impact of injection-water salinity and ionic content on oil recovery from carbonate reservoirs. SPE Reserv. Eval. Eng..

[30] T. Qin, P. Fenter, M. AlOtaibi, and S. Ayirala, in *SPE Improved Oil Recovery Conference, Virtual, April 2022.* Virtual, (2022), pp. SPE.

[31] Fenter PA (2002). X-ray reflectivity as a probe of mineral-fluid interfaces: A user guide. Rev. Mineral. Geochem..

[32] Robinson IK (1998). X-ray crystallography of surfaces and interfaces. Acta Crystallogr. A.

[33] Feidenhans'l R (1989). Surface-structure determination by X-ray-diffraction. Surf. Sci. Rep..

