# Supplementary material for: Systematic observations of enhanced oil recovery and associated changes at carbonate-brine and carbonate-petroleum interfaces
Source: Sci Rep. 2023 Oct 6;13:16891. doi: 10.1038/s41598-023-43081-2 (PMC10558449; doi:10.1038/s41598-023-43081-2)
Supplement: Supplementary file 1 — Supplementary Tables. [file 41598_2023_43081_MOESM1_ESM.docx]

Supplementary information for:

**Systematic Observations of Enhanced Oil Recovery and Associated Changes at Carbonate-Brine and Carbonate-Petroleum Interfaces**

Tianzhu Qin^†^, Paul Fenter^*^

Chemical Sciences and Engineering Division, Argonne National Laboratory

Mohammed AlOtaibi, Subhash Ayirala, Ali Yousef

EXPEC Advanced Research Center, Saudi Aramco

*Corresponding Author: fenter@anl.gov

^†^Current address: Sigray Corporation, Concord CA 94520

**Supplemental Table 1:** Parameters that describe the calcite-water interfaces for calcite saturated solution and the TW2 brine.

**Supplemental Table 2:** Parameters that describe the calcite-petroleum interface, the calcite-TW2 interface (after displacement of the petroleum), and the calcite-petroleum interface (after displacement of the TW2 brine).
